# Effectiveness of plyometric training vs. complex training on the explosive power of lower limbs: A Systematic review

**DOI:** 10.3389/fphys.2022.1061110

**Published:** 2023-01-18

**Authors:** Xiaolin Wang, Changhai Lv, Xinmin Qin, Shuyu Ji, Delong Dong

**Affiliations:** ^1^ Department of Sport Studies, Faculty of Educational Studies, University Putra Malaysia, Serdang, Selangor, Malaysia; ^2^ Department of Physical Education, Shandong Technology and Business University, Yantai, Shandong, China; ^3^ Department of Sport Science, Kangwon National University, Chuncheon, South Korea; ^4^ Faculty of Educational Studies, Taizhou University, Taizhou, Zhejiang, China; ^5^ Department of Physical Education, Ludong University, Yantai, Shandong, China

**Keywords:** plyometric exercises, complex exercises, resistance training, explosive force, muscular strength

## Abstract

**Introduction:** Explosive power is considered an important factor in competitive events. Thus, strategies such as complex training (CT) and plyometric training (PLT) are effective at improving explosive power. However, it is still not clear which of the two strategies can enable greater improvements on the explosive power. Thus, the aim of this systematic review was to compare the effects of PLT and CT on the explosive power of the lower limbs.

**Methods:** The Review Manager and GraphPad Prism programs were used to analyze the synthetic and time effects (effects over training time) on explosive power (i.e., jump ability, sprint ability) and maximum strength. Our research identified 87 studies comprising 1,355 subjects aged 10–26.4 years.

**Results:** The results suggested the following: 1) Synthetic effects on jump ability (Hedges’ *g*): .79 (*p* < .001) for unloaded PLT, 1.35 (*p* < .001) for loaded PLT and .85 (*p* < .001) for CT; 2) Synthetic effects on sprint ability: .83 (*p* < .001) for unloaded PLT, −2.11 (*p* < .001) for loaded PLT and −.78 (*p* < .001) for CT; 3) Synthetic effects on maximum strength: .84 (*p* < .001) for loaded PLT and 1.53 (*p* < .001) for CT; 4) The time effects of unloaded PLT and CT on explosive power were similar, but the time effects of CT on maximum strength were obviously above that of PLT.

**Discussion:** In conclusion, unloaded PLT and CT have a similar effect on explosive performance in the short term but loaded PLT has a better effect. The improvement of the maximum strength caused by CT was greater than that induced by PLT. In addition, more than 10 weeks of training may be more beneficial for the improvement of power. Therefore, for explosive power training, we suggest adopting unloaded or light-loaded PLT during a short season and applying CT during an annual or long training cycle.

## Introduction

Explosive power is the ability to exert great muscular strength in a very short time (usually within 100 m) (Maffiuletti et al., 2016), which is vital for athletes to win the competition. Therefore, coaches and researchers have been concerned with how to select more efficient explosive training methods. Traditional resistance training (RT), plyometric training (PLT) and complex training (CT) have been widely used in strength and explosive training. The three training methods have different effects on explosive power. Traditional resistance training (RT) can increase athletes’ maximum strength but does not greatly increase explosive power because of the sticking point (Kompf and Arandjelović, 2016). When the joint reaches a certain angle in resistance motion, the muscle force will be minimized, and the movement speed will decrease, called the sticking zone (Kompf and Arandjelović, 2016). The reduction of force and speed is not conducive to the development of explosive force. Plyometric training (PLT), which follows the form of human movement, uses the principle of the “stretch-shortening cycle” (SSC) to transform the elastic potential energy in the eccentric contraction stage into kinetic energy in the concentric contraction stage (Ramirez-Campillo et al., 2020). It has a significant effect on the improvement of explosive power, but the low-load characteristic limits the improvement of maximum strength, and then the maximum force further limits the development of explosive power (Suchomel et al., 2016). Therefore, PLT may not be conducive to the long-term development of explosive power. Complex training (CT) combining RT and PLT uses postactivation potentiation (PAP) and stretch-shortening cycle (SSC) to enhance the explosive power and maximum strength of athletes (Carter and Greenwood, 2014) and seems to be a good choice for the improvement of explosive power. However, does CT improve explosive power better than PLT?

PLT and CT have better effects on the improvement of explosive power than TR (Behm et al., 2017; Pardos-Mainer et al., 2021; Morris et al., 2022). However, academic circles have not reached a consensus on whether CT is better than PLT in improving explosive power. Theoretically, CT integrates the advantages of PLT for the increasement of explosive power and RT for the enhancement of maximum strength, so as to improve the explosive power better. However, according to the force-velocity curve, there is an inverse relationship exists between force and velocity (Taber et al., 2016). Therefore, it is hard to improve both strength and speed (i.e., explosive power). Studies by Hammami et al. (2019); Zghal et al. (2019) showed that CT had better effects on improving jump ability, sprint ability and maximum strength than PLT, but some other studies showed that these two training methods had no significant difference in the improvement of jump performance (Arabatzi et al., 2010; da Silva Santos et al., 2015; Sánchez-Sixto et al., 2021). Lloyd et al. (2016) reported that there was no significant difference between these two training methods in improving the sprint ability of teenagers. We learned from previous research that PLT and CT have a significant effect on the improvement of explosive power. However, there is still great controversy concerning which training method is better.

Due to limited comparative studies (CT vs PLT), there have been no meta-analysis reviews conducted regarding the comparative effects of PLT and CT on explosive power. However, we used data from different experiments to conduct a meta-analysis by referring to Behm’s analysis method (Behm et al., 2017). In addition, we also compared the changes of the average effect size of the CT and PLT on explosive performance over time in order to display the research results more clearly. Therefore, the intent of this systematic review was to compare the effects of PLT and CT on explosive power. Because explosive power is strongly correlated with maximum strength (Taber et al., 2016), we also aim to compare the effects of CT and PLT on maximum strength. Another purpose was to provide clear guidelines for the prescription of explosive power training.

## Methods

### Experimental approach to the problem

This systematic review was conducted according to the Cochrane Collaboration Guidebook and the criteria of Preferred Reporting Elements for Systematic Reviews and Meta-analyses (Liberati et al., 2009).

## Search strategy

Articles published by 10 June 2022, were located using the electronic databases PubMed, SCOPUS, SPORTDiscus, and Web of Science. The following search syntax was used (“randomized controlled trial” OR “controlled clinical trial”) [Publication Type] (“plyometric” OR “stretch-shortening cycle” OR “jump” OR “power” OR “complex” OR “compound” OR “combined”) AND (“training” OR “intervention”) [Title/Abstract]. Lead author’s personal libraries and gray literature sources (e.g., conference proceedings) were also examined. The systematic search process was conducted by JS and QX. Any disagreement of an included/excluded study was resolved by the third author (WX).

### Eligibility Criteria

The inclusion criteria were as follows: 1) the study was randomized controlled trial or controlled trial. 2) The study employed a PLT or CT intervention inclusive of 2‒3 related exercises per week and more than a 4-week training duration. 3) The related indices of explosive power and maximum strength before and after training were measured in the study. The explosive power indices included jump ability (countermovement jump, squat jump, long jump, etc.) and sprint ability (10 m sprint or close distance sprint). The maximum strength indices included the one-repetition maximum (full squat, half squat, leg flexion and extension, etc.). 4) The subjects were healthy athletes or students with training experience, and their ages ranged from 10 to 30. Since this meta-analysis is not for comparative studies of the same experiment, sufficient sample size must be ensured to reduce errors. Therefore, we chose a wide age range (teenagers and young adults).

### Exclusion criteria

**FIGURE 1:**
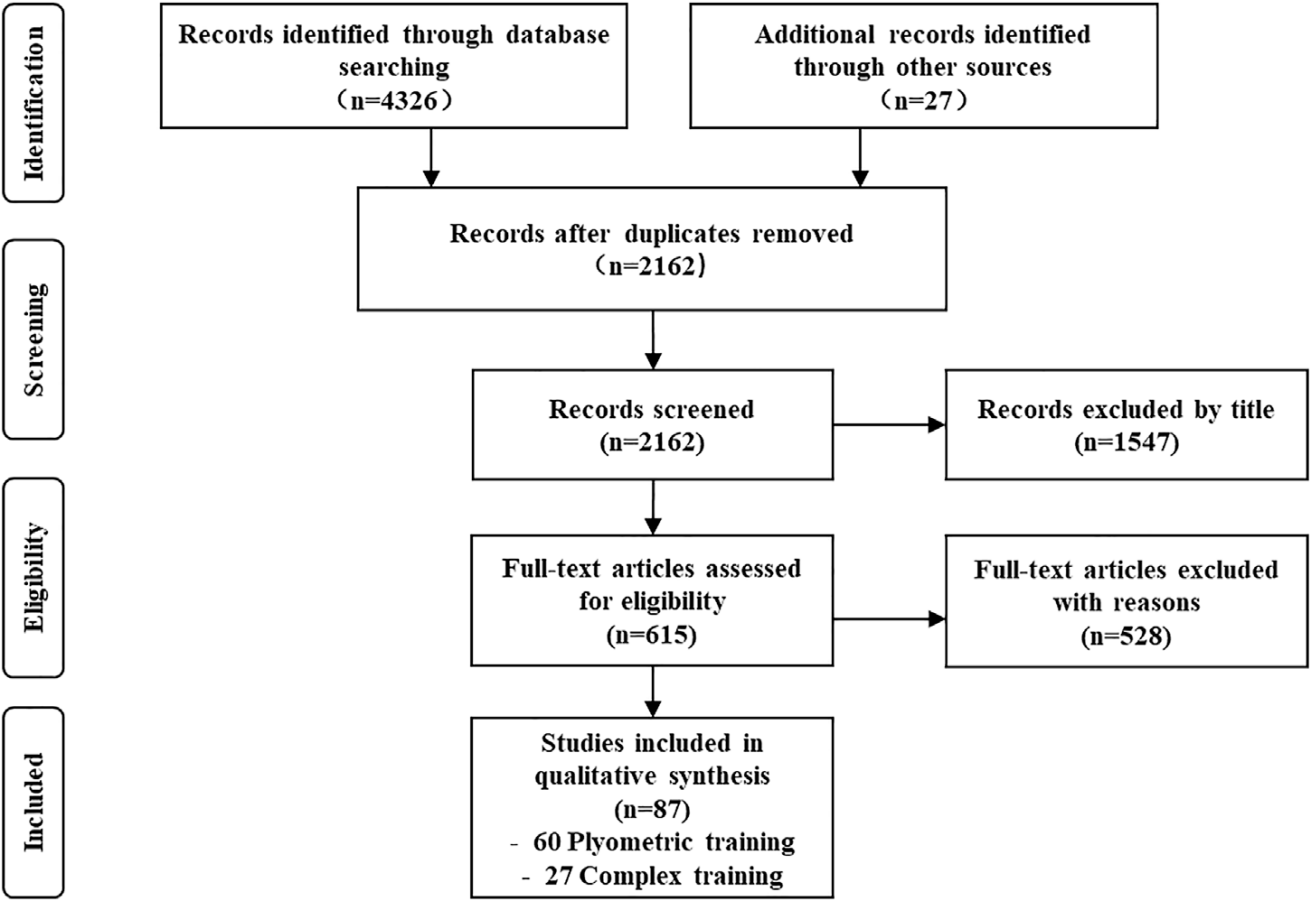
Flowchart of study selection (PLT = plyometric training, CT = complex training).

Excluded records had the following: a) not available in English; b) non-human experiment; c) cross-sectional study; d) meta-analysis or review article; e) lack of data to calculate effect size (i.e., sample size, mean and standard deviation); and f) overtraining or distraining study. The screening flow chart is shown in Figure 1.

### Data extraction

Data extraction from the included studies was independently performed by two authors (S.J. and X.M.). We extracted data from PLT- and CT-related studies. The following data were extracted: the first author’s name, publication year, sample size, subjects’ age (years), sex, training duration (weeks), training program, and outcome indicators (details in the Eligibility Criteria section). In instances where information was unavailable for the mean and standard deviation, lead authors were contacted for data. If no answer was received, the study was excluded. Any disagreement in data extraction was resolved by the third author (WX).

### Statistical analyses

The Physiotherapy Evidence Database (PEDro) scale was used to assess the risk of all included studies. There are 11 items in the PEDro checklist for a total of 10 points (item 1 is not rated). As in a similar previous plyometric training meta-analysis, literature quality was interpreted as “low quality” (≤3 points), “medium quality” (4‒5 points), or “high quality” (6–10 points) (Stojanović et al., 2017). The results of the literature evaluation included in this study are shown in Table 1 and Table 2.

**TABLE 1:** Characteristics of analyzed PLT studies and PEDro scores.

Article	N	G	W	Outcomes	PEDro scale (items*)											T
					a	b	c	d	e	f	g	h	i	j	k	
Aloui et al. (2021)	17	M	8	CMJA, SQJ, 10–30 m SP	1	1	0	1	0	0	0	1	1	1	1	6
Arabatzi et al. (2010)	9	M	8	CMJ, SQJ	1	1	0	0	0	0	0	1	1	1	1	5
Asadi et al. (2016)	8	M	8	VJ, 60 m SP	1	0	0	0	0	0	0	1	1	1	1	4
Asadi et al. (2018)	30	M	6	VJ, 20 m SP, PF	1	0	0	0	0	0	0	1	1	1	1	4
Blattner and Noble (1979)	11	M	8	VJ	1	1	0	0	0	0	0	1	1	1	1	5
Campo et al. (2009)	10	F	12	CMJ, DJ	1	1	0	1	0	0	0	1	1	1	1	6
Chelly et al. (2010)	12	M	8	CMJ, SQJ	1	1	0	0	0	0	0	1	1	1	1	5
Chelly et al. (2015)	14	M	10	SQJ, CMJ, PF	1	1	0	1	0	0	0	1	1	1	1	6
Chelly et al. (2014)	12	M	8	CMJ, DJ, PF	1	1	0	0	0	0	0	1	1	1	1	5
Cherni et al. (2021)	15	F	8	CMJ, SQJ, 10–30 m SP, PF	1	0	0	0	0	0	0	1	1	1	1	4
Chtara et al. (2017)	10	M	6	10, 30 m SP	1	1	0	1	0	0	0	1	1	1	1	6
Coratella et al. (2018)	32	M	8	CMJ,10, 30 m SP, 1 RM SQ	1	1	0	0	0	0	0	1	1	1	1	5
Drouzas et al. (2020)	23	M	9	CMJ,5–20 m SP, PF	1	1	0	0	0	0	0	0	1	1	1	4
Fathi et al. (2019)	20	M	16	CMJ, SQJ, 5, 10 m SP	1	1	0	0	0	0	0	0	1	1	1	4
Fernandez-Fernandez et al. (2016)	30	M	8	CMJ, 5, 10, 20 m SP	1	0	0	1	0	0	0	1	1	1	1	5
Gehri et al. (1998)	18	C	12	SQJ, CMJ, DJ	1	1	0	0	0	0	0	1	1	1	1	5
González-Ravé et al. (2019)	12	M	3	CMJ, SQJ	1	1	0	0	0	0	0	1	1	1	1	5
Hall et al. (2016)	10	F	6	CMJ	1	1	0	1	0	0	0	1	1	1	1	6
Hammami et al. (2016)	15	M	8	5–40 m SP	1	1	0	0	0	0	0	1	1	1	1	5
Hammami et al. (2017a)	14	M	8	CMJ, SQJ, 5–40 m SP, 1 RM SQ	1	1	0	1	0	0	0	1	1	1	1	6
Hammami et al. (2020b)	17	F	10	5–30 m SP	1	1	0	1	0	0	0	1	1	1	1	6
Hammami et al. (2020a)	26	M	10	5–40 m SP, CMJ	1	1	1	0	1	1	1	1	1	1	1	9
Houghton et al. (2013)	7	M	8	5 m SP, CMJ, SQJ, PF	1	0	0	0	0	0	0	1	1	1	1	4
Dello Iacono et al. (2017)	9	M	10	10, 20 m S, CMJ	1	0	0	1	0	0	0	1	1	1	1	5
Idrizovic et al. (2018)	13	F	12	CMJ	1	1	0	0	0	0	0	1	1	1	1	5
Jlid et al. (2019)	14	M	8	SQJ, CMJ	1	1	0	1	0	0	0	1	1	1	1	6
Jlid et al. (2020)	14	M	6	CMJ, SQJ	1	1	0	1	0	0	0	1	1	1	1	6
Karadenizli (2016)	14	F	10	CMJA, 30 m SP	1	1	0	0	0	0	0	1	1	1	1	5
Kato et al. (2006)	18	F	24	VJ	1	0	0	0	0	0	0	1	1	1	1	4
Khlifa et al. (2010)	18	M	10	CMJ, SQJ	1	1	0	0	0	0	0	1	1	1	1	5
Khodaei et al. (2017)	8	M	4	10, 30 m SP	1	1	0	0	0	0	0	0	1	1	1	4
Kobal et al. (2017)	20	M	6	CMJ, SQJ	1	0	0	0	0	0	0	1	1	1	1	4
Maciejczyk et al. (2021)	7	F	4	CMJ, SQJ	1	1	1	0	0	0	0	1	1	1	1	6
Makaruk et al. (2014)	24	M	6	CMJ, DJ	1	1	0	1	0	0	0	1	1	1	1	6
Mazurek et al. (2018)	14	M	5	CMJ, DJ, SQJ	1	1	0	0	0	0	0	1	1	1	1	5
McKinlay et al. (2018)	13	M	8	CMJ, SQJ	1	0	0	1	0	0	0	1	1	1	1	5
Meszler and Váczi (2019)	9	F	7	CMJ	1	1	0	1	0	0	0	1	1	1	1	6
Negra et al. (2017)	18	M	8	CMJ, 10, 20, 30 m SP	1	1	0	0	0	0	0	1	1	1	1	5
Negra et al. (2020a)	13	M	8	20 m S, DJ (20, 40 cm)	1	1	0	1	0	0	0	1	1	1	1	6
Negra et al. (2020b)	11	M	12	CMJ, SQJ, 20 m SP, 1 RM SQ	1	1	0	0	0	0	0	1	1	1	1	5
Negra et al. (2020)	13	M	8	CMJ, 5, 10, 20 m SP	1	1	0	1	0	0	0	1	1	1	1	6
Ozbar et al. (2014)	9	F	8	CMJ, 20 m SP	1	0	0	0	0	0	0	1	1	1	1	4
Ozbar (2015)	10	F	10	10, 20, 30 m SP, CMJ	1	0	0	1	0	0	0	1	1	1	1	5
Palma-Muñoz et al. (2021)	15	M	6	CMJ, DJ, 10 m SP	1	0	0	0	0	0	0	1	1	1	1	4
Pereira et al. (2015)	10	F	8	CMJ	1	1	0	1	0	0	0	1	1	1	1	6
Ramírez-Campillo et al. (2016)	40	C	6	CMJ, DJ, 30 m SP	1	1	1	1	0	0	0	1	1	1	1	7
Ramirez-Campillo et al. (2018)	16	F	8	CMJ, DJ, 15 m SP	1	1	1	1	0	0	0	0	1	1	1	6
Ramirez-Campillo et al. (2019)	19	M	7	CMJ, 20 m SP	1	1	0	0	0	0	0	1	1	1	1	5
Rosas et al. (2017)	8	F	6	CMJ, SQJ, 20 m SP	1	1	0	1	0	0	0	1	1	1	1	6
Rubley et al. (2011)	10	F	12	VJ	1	0	0	0	0	0	0	1	1	1	1	4
Sammoud et al. (2021)	12	F	8	CMJ	1	1	1	1	0	0	0	1	1	1	1	7
Sánchez-Sixto et al. (2021)	11	F	6	VJ, PF	1	0	0	0	0	0	0	1	1	1	1	4
Söhnlein et al. (2014)	12	M	16	10, 20, 30 m SP	1	0	0	0	0	0	0	1	1	1	1	4
Teo et al. (2016)	13	M	6	CMJ, SQJ, DJ, 20 m SP	1	1	1	0	0	0	0	1	1	1	1	6
Váczi et al. (2013)	12	M	6	DJ, PF	1	1	0	0	0	0	0	1	1	1	1	5
Vera-Assaoka et al. (2020)	38	M	7	CMJ, DJ, 20 m SP	1	1	0	0	0	0	0	1	1	1	1	5
de Villarreal et al. (2015)	13	M	9	CMJ, 5, 10 m SP	1	1	0	0	0	0	0	1	1	1	1	5
Whitehead et al. (2018)	10	M	8	VJ, 20 m SP	1	0	0	1	0	0	0	1	1	1	1	5
Wilson et al. (1996)	14	M	8	CMJ, PF	1	1	0	1	0	0	0	1	1	1	1	6
Yanci et al. (2017)	27	M	6	5, 15 m SP, CMJ	1	1	1	0	0	0	0	1	1	1	1	6
Young et al. (1999)	24	M	6	SQJ, PF	1	1	0	0	0	0	0	0	1	1	1	4
Zghal et al. (2019)	9	M	7	CMJ, SQJ, 5–20 m SP, PF	1	1	0	1	0	0	0	1	1	1	1	6
Zribi et al. (2014)	25	M	9	CMJ, SQJ	1	1	0	0	0	0	0	1	1	1	1	5

Note: Abbreviations descriptions are ordered alphabetically. C, combination; CMJ, countermovement jump; DJ, depth jump; F, female; G, gender; M, male; N, number of subjects; PF, peak force; 1 RM, one-repetition maximum; RT, resistance training; SP, sprint; SQ, squat; SQJ, squat jump; T, total scores; VJ, vertical jump; W, weeks (training duration); item* a-k detailed explanation for each PEDro scale item can be accessed at https://pedro.org.au/wp-cotent/uloads/PEDro_scale.pdf (access for this review: 26 August 2022). More detailed data are available on the website https://drive.google.com/drive/folders/1b72Elsw-GTtCpQ0sxprwshisEjRSKlZB.

**TABLE 2:** Characteristics of analyzed CT studies and PEDro scores.

Study	N	G	W	Outcomes	PEDro (items*)											T
					a	b	c	d	e	f	g	h	i	j	k	
Ali et al. (2019)	12	M	6	CMJ, 20 m SP	1	1	1	1	0	0	0	1	1	1	1	7
Alves et al. (2010)	17	M	6	CMJ, SQJ, 5, 20 m SP	1	0	0	0	0	0	0	1	1	1	1	4
Arabatzi et al. (2010)	10	?	8	CMJ, SQJ	1	1	0	0	0	0	0	1	1	1	1	5
Carlson et al. (2009)	9	C	6	CMJ	1	1	0	0	0	0	0	1	1	1	1	5
Daehlin et al. (2016)	9	M	8	LJ, 1 R M SQ	1	1	0	0	0	0	0	1	1	1	1	5
Faigenbaum et al. (2007)	13	M	6	VJ, 9.1 m SP	1	0	0	1	0	0	0	1	1	1	1	5
Fathi et al. (2019)	20	?	16	CMJ, SQJ, 5, 10 m SP	1	1	0	0	0	0	0	0	1	1	1	4
Fatouros et al. (2000)	10	M	10	CMJ, 1 R M SQ	1	1	0	0	0	0	0	1	1	1	1	5
Faude et al. (2013)	8	M	7	CMJ, 10 m SP, 1 RM SQ	1	1	0	0	0	0	0	1	1	1	1	5
Franco-Márquez et al. (2015)	20	M	6	CMJ, 1 RM SQ	1	0	0	0	0	0	0	1	1	1	1	4
Hammami et al. (2017b)	16	M	8	CMJ, 20 m SP, 1 R M SQ	1	1	0	0	0	0	0	1	1	1	1	5
Hammami et al. (2017a)	19	M	8	5 m SP, 1 RM 1/2 SQ	1	1	0	0	0	0	0	1	1	1	1	5
Hammami et al. (2019)	14	M	8	CMJ, SQJ, 40 m SP, SQ	1	1	0	1	0	0	0	1	1	1	1	6
Kijowksi et al. (2015)	9	M	4	CMJ, 1 R M SQ	1	1	0	0	0	0	0	1	1	1	1	5
Li et al. (2019)	10	M	8	CMJ, 50 m SP, 1 R M SQ	1	0	0	0	0	0	0	1	1	1	1	4
Lloyd et al. (2016)	20	M	6	SQJ, 10, 20 m SP	1	0	0	0	0	0	0	1	1	1	1	4
Lyttle et al. (1996)	11	M	8	CMJ, 40 m SP, 1 RM SQ	1	1	0	0	0	0	0	1	1	1	1	5
Rodríguez-Rosell et al. (2016)	15	?	6	CMJ, 10 m SP, 1 RM SQ	1	1	0	1	0	0	0	1	1	1	1	6
Rodríguez-Rosell et al. (2017)	10	?	6	CMJ, 10 m SP, 1 RM SQ	1	1	0	1	0	0	0	1	1	1	1	6
Latorre Román et al. (2018)	30	C	10	CMJ, SQJ, 25 m SP	1	1	0	0	0	0	0	1	1	1	1	5
Sánchez-Sixto et al. (2021)	13	F	10	CMJ, PF	1	0	0	0	0	0	0	1	1	1	1	4
Santos and Janeira (2008)	15	M	10	CMJ, SQJ	1	1	0	0	0	0	0	1	1	1	1	5
Talpey et al. (2016)	9	M	9	CMJ, 20 m SP, 1 RM SQ	1	1	0	1	0	0	0	1	1	1	1	6
Veliz et al. (2014)	16	?	18	CMJ, 1 RM SQ	1	1	0	1	0	0	0	1	1	1	1	6
Veliz et al. (2015)	11	F	16	CMJ, 1 RM SQ	1	1	0	1	0	0	0	1	1	1	1	6
Voelzke et al. (2012)	8	?	5	5 m SP	1	1	0	1	0	0	0	1	1	1	1	6
Zghal et al. (2019)	14	M	7	CMJ, SQJ, 10 m SP, PF	1	1	0	1	0	0	0	1	1	1	1	6

Note: Abbreviations descriptions are ordered alphabetically. C, combination; CMJ, countermovement jump; DJ, depth jump; F, female; G, gender; M, male; N, number of subjects; PF, peak force; 1 RM, one-repetition maximum; RT, resistance training; SP, sprint; SQ, squat; SQJ, squat jump; T, total scores; VJ, vertical jump; W, weeks (training duration); item* a-k detailed explanation for each PEDro scale item can be accessed at https://pedro.org.au/wp-cotent/uloads/PEDro_scale.pdf (access for this review: 26 August 2022). More detailed data are available on the website https://drive.google.com/drive/folders/1UhXv4vrrqcFcll9RthGEAfDPafcvB22M.

Effect sizes (Hedges’ *g*), standard error and 95% confidence interval were calculated by mean value and standard deviation. The pre-and post-experimental data of the experimental group were used for the analysis and calculation because of baseline differences between the experimental and control groups in the same study and differences in physical activity in control groups of different studies. Calculated effect sizes were interpreted using the following scale: .2–.5 = small, .5–.8 = moderate, > .8 = large (Stojanović et al., 2017). The effect sizes are usually positive, but sprint ability has negative effect sizes. This is because better sprint ability means less time to complete the same distance. The Review Manager Software (RevMan 5.3) was used to analyze the synthetic effects of PLT and CT on mechanistic changes (i.e., jump ability, sprint ability, and maximum strength), as well as the synthetic effects of the PLT subgroup (i.e., unloaded PLT and loaded PLT). We used the random-effects model to avoid the high weight of individual studies affecting the overall synthetic effect. In addition, GraphPad Prism Software (GraphPad Prism 8) was used to perform a linear fitting of mechanistic changes with time and compare the difference in the time effect between PLT and CT. Due to the limited data of loaded PT, we only compared the difference in the time effect between unloaded PLT and CT in this study.

## Results

### Study selection, characteristics, and risk of bias

In total, 60 PLT studies and 27 CT studies were included in this study, producing 143 and 63 effect sizes, respectively (Table 3). The included studies involved 996 PLT participants and 359 CT participants, with an average age range of 10–24.2 years and 9.7–26.4 years, respectively. The PLT program mainly included countermovement jump, squat jump, depth jump, long jump, hurdle jump, single- or double-legged jump, etc., and its training load was usually body weight and light weight (less than 25% extra body weight). The CT program mainly combines traditional resistance training (RT) (e.g., back squat, half squat, calf raise, leg flexion and extension, snatch and weight lifting, etc.) and PLT program. The load intensity of RT is usually 40%–95% of 1 RM. The shortest training duration was 4 weeks, and the longest training durations of PLT and CT were 24 weeks and 18 weeks, respectively. Among the included studies, most studies achieved 5‒6 points (medium-high quality), with the highest score achieving 9 points and the lowest score achieving 4 points. The PEDro scale score had a median of 5 of 10 points across studies (Tables 1, 2).

**TABLE 3:** Training participants and effects characteristics.

Training methods	No. of studies	No. of subjects	No. of effects	No. of effects for JP	No. of effects for SP	No. of effects for MS
PLT	60	996	143	71	46	16
CT	27	359	63	26	19	18

Note: JP, jump performance; SP, sprint performance; MS, maximum strength.

### Synthetic results

**FIGURE 2:**
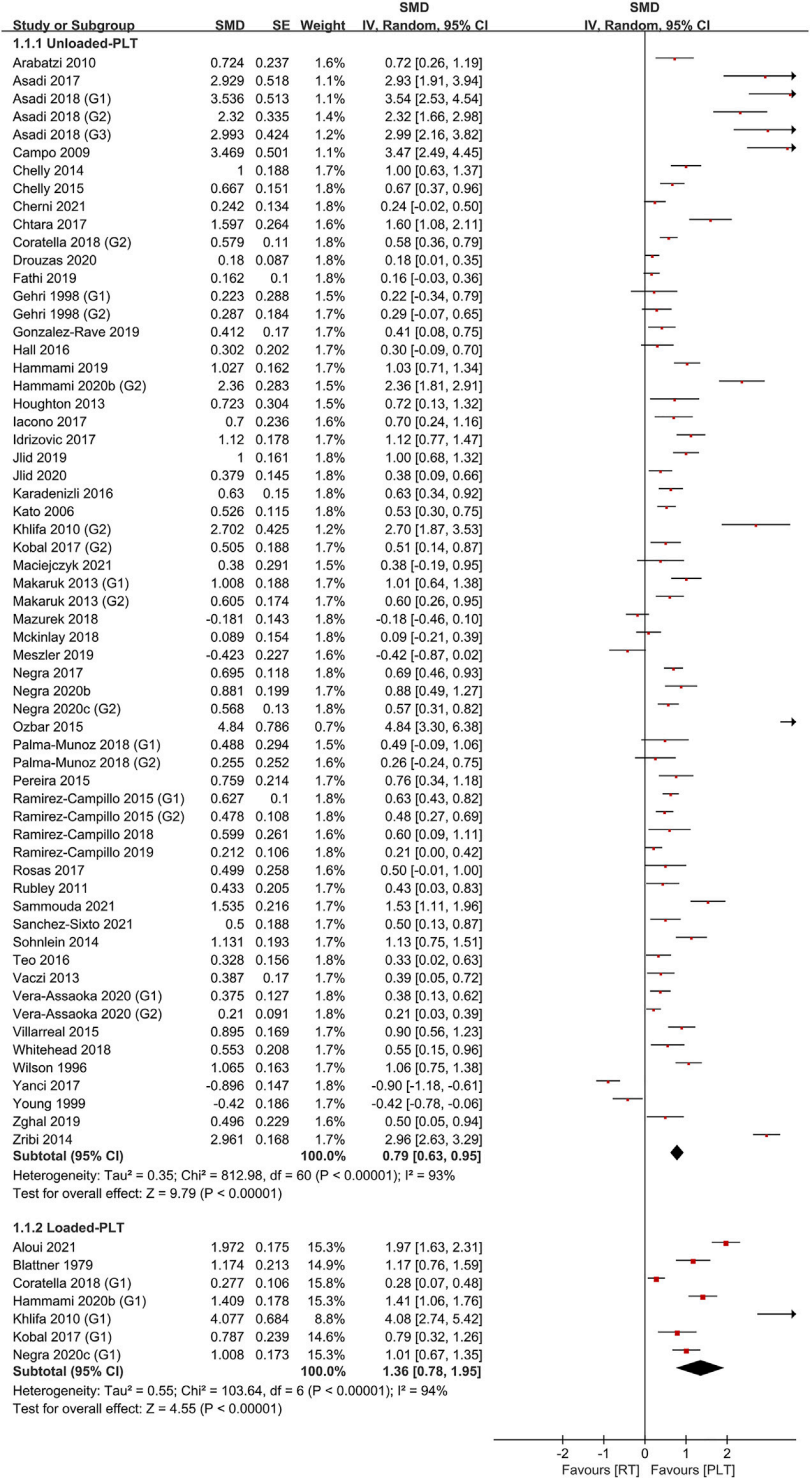
Effects of plyometric training (PLT) on jump performance.

**FIGURE 3:**
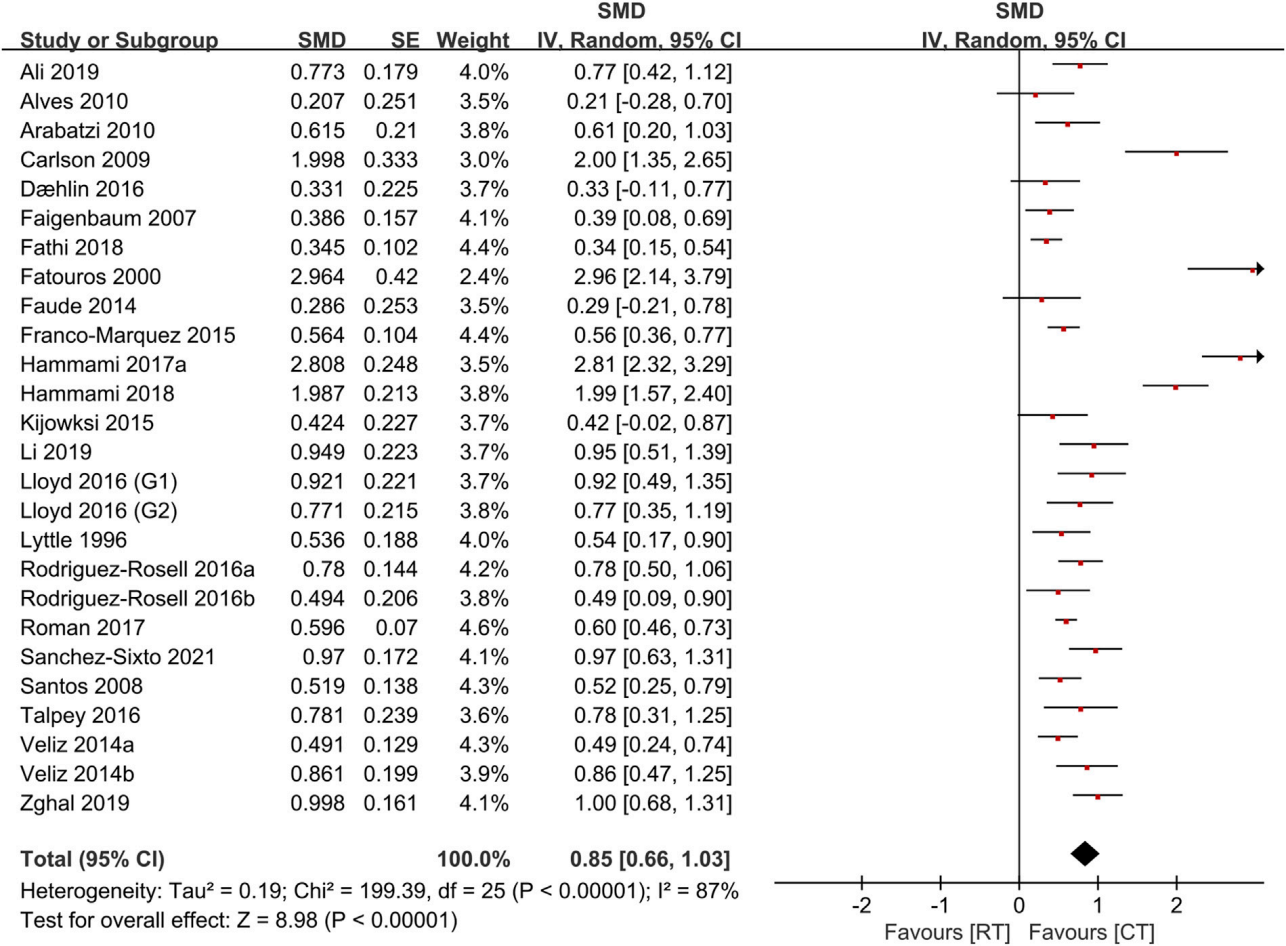
Effects of complex training (PLT) on jump performance.

**FIGURE 4:**
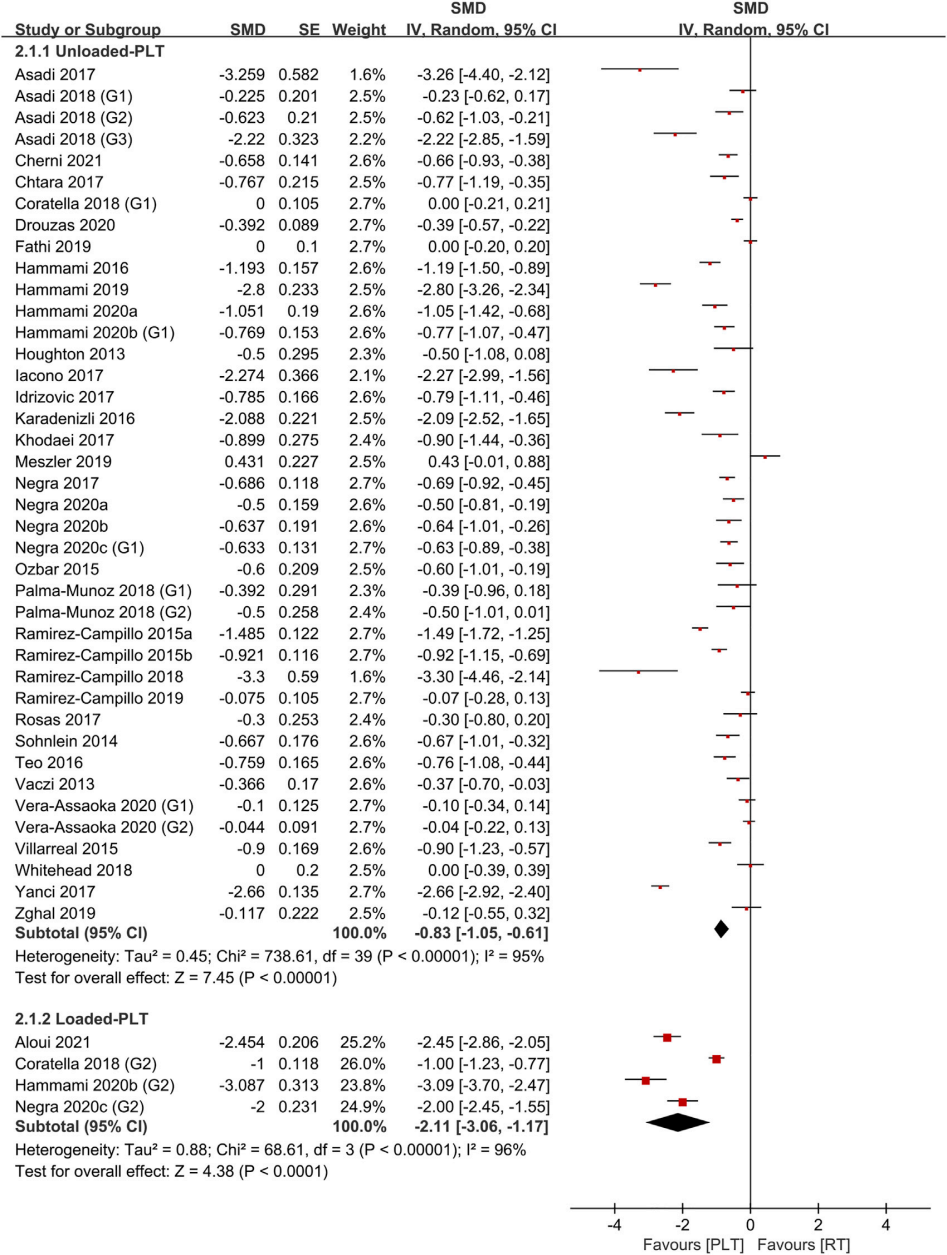
Effects of plyometric training (PLT) on sprint performance.

**FIGURE 5:**
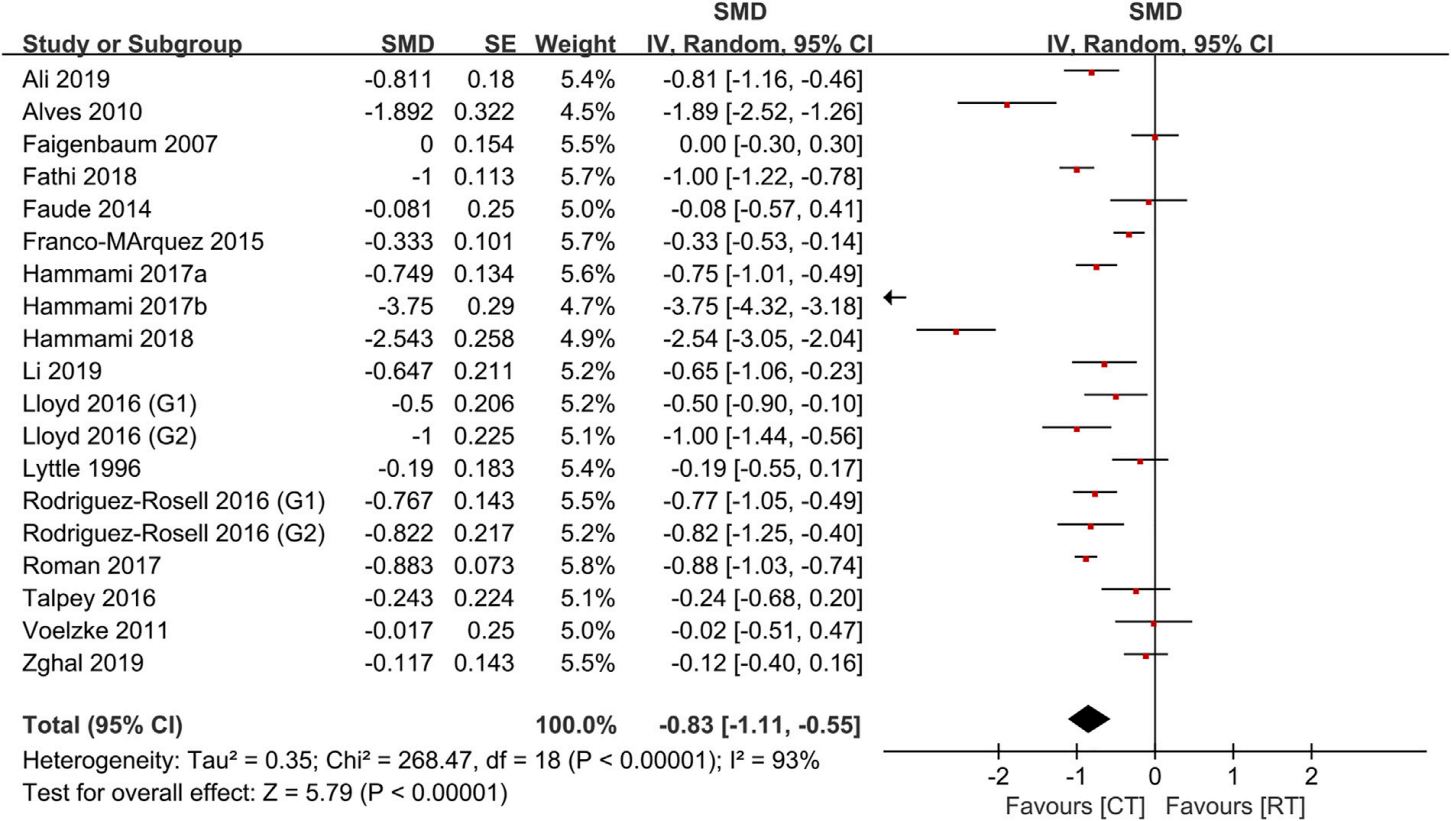
Effects of complex training (PLT) on sprint performance.

**FIGURE 6:**
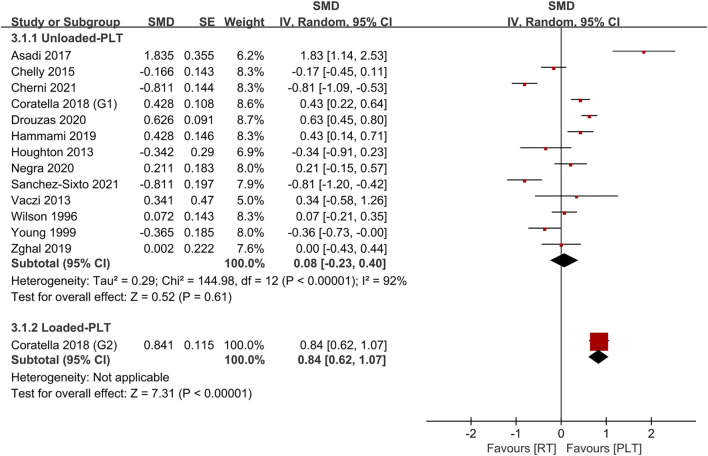
Effects of plyometric training (PLT) on maximum strength.

**FIGURE 7:**
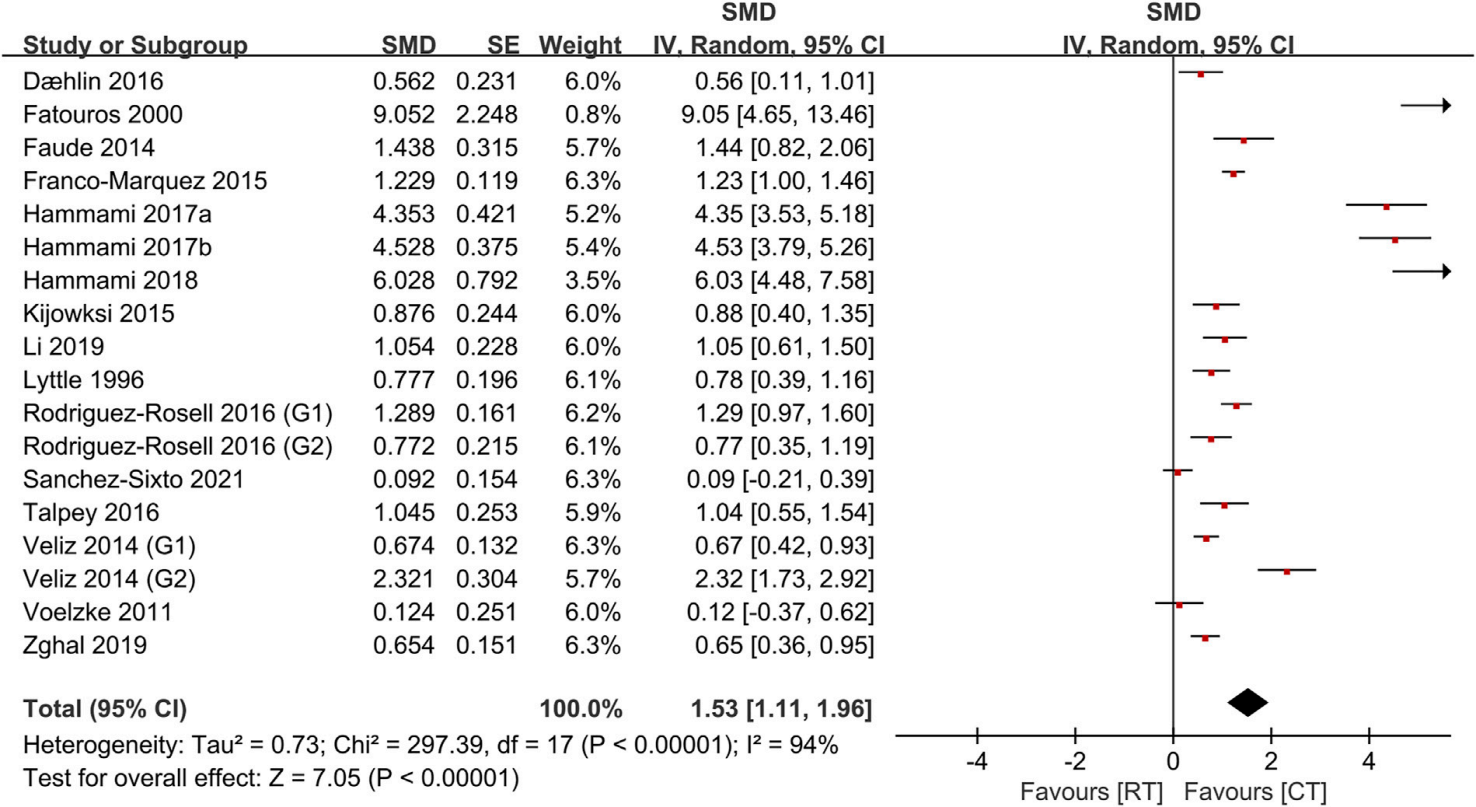
Effects of complex training (PLT) on maximum strength.

Table 4 shows the comparative effects of PLT and CT on jump ability, sprint ability and maximum strength. In terms of effect sizes on jump ability, CT studies slightly exceeded unloaded PLT studies (.85, *p* < .001, large vs .79, *p* < .001, medium), but the loaded PLT showed the largest effect size (1.35, *p* < .001, large) (Figures 2, 3). In terms of effect sizes on sprint ability, unloaded PLT studies and CT studies showed the same effect size (−.83, *p* < .001, large), but the effect size of loaded PLT studies (−2.11, *p* < .001, large) was larger than that of unloaded PLT and CT studies (Figures 4, 5). In terms of effect sizes on maximum strength, both loaded and unloaded PLT studies were lower than CT studies, especially unloaded PLT studies with an effect size of .08 (Figures 6, 7).

**TABLE 4:** Summary of meta-analysis results.

Effect size	CT	Unloaded PLT	Loaded PLT
Jump ability	.85 *** Large	.79 *** Moderate	1.35 *** Large
Sprint ability	−.83 *** Large	−.83 *** Large	−2.11*** Large #
Maximum strength	1.53 *** Large	.08 *** trivial	.84 *** Large ##

Note: # 4 studies met inclusion criteria, ## 1 study met inclusion criteria. ***p < .001.

### Time effect fitting results

**FIGURE 8:**
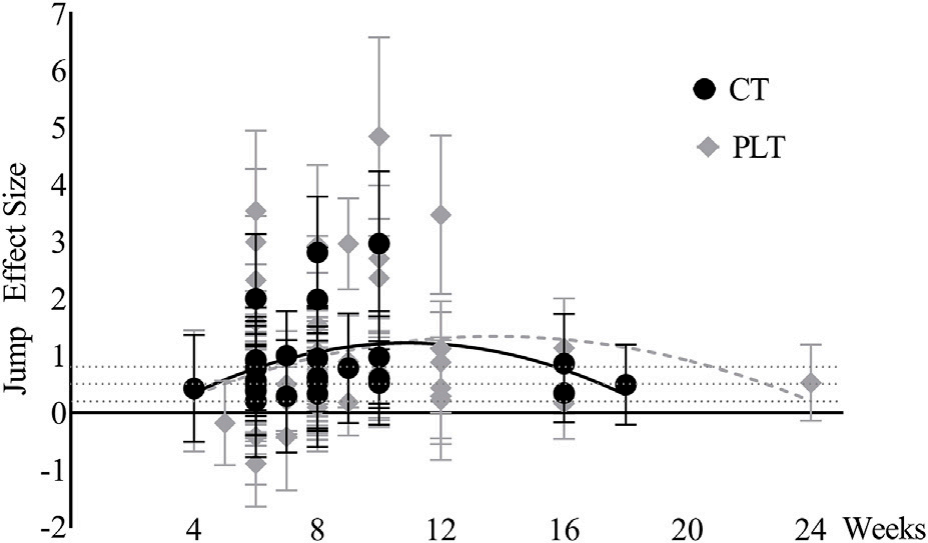
Jump ability changes over time (CT = complex training, PLT = plyometric training).

Time effects on jump ability. The time effects of PLT and CT on jump ability both increased at first and then decreased (Figure 8). Due to limited data after 12 weeks, we did not take into account trends thereafter. Before 10 weeks, the curves of the effect size of PLT and CT on jump ability were basically the same, with a large effect at week 6 for both, the peak effect at week 10 for CT and approximately week 10–12 for PLT.

**FIGURE 9:**
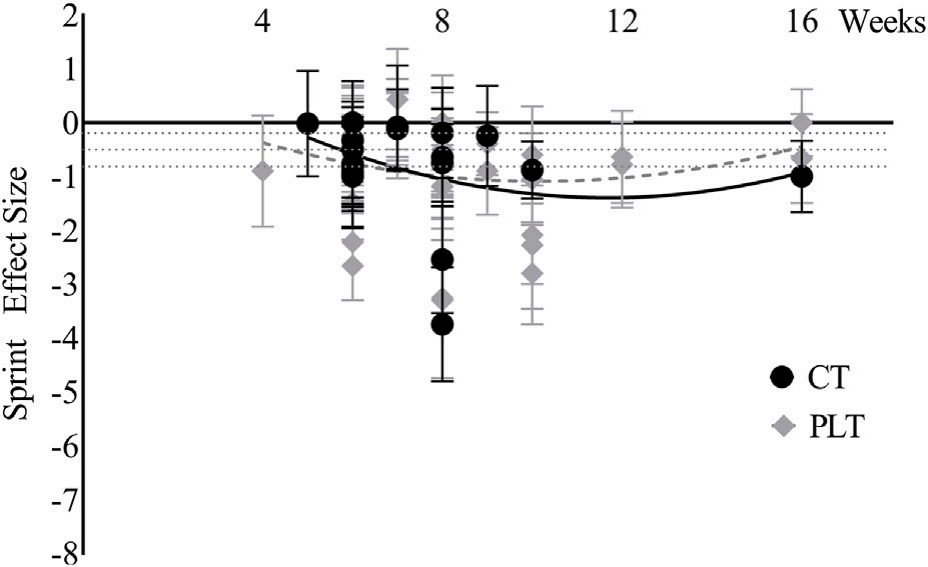
Sprint ability changes over time (CT = complex training, PLT = plyometric training).

Time effects on sprint ability. The curves of the effect size of PLT and CT on sprint ability are similar to those on jump ability (Figure 9). Before 10 weeks, the trends of the effect size of PLT and CT on sprint ability were basically the same, with a moderate effect at week 6 and a large effect at week 8 for both, a peak effect at week 8 for CT and week 10 for PLT.

**FIGURE 10:**
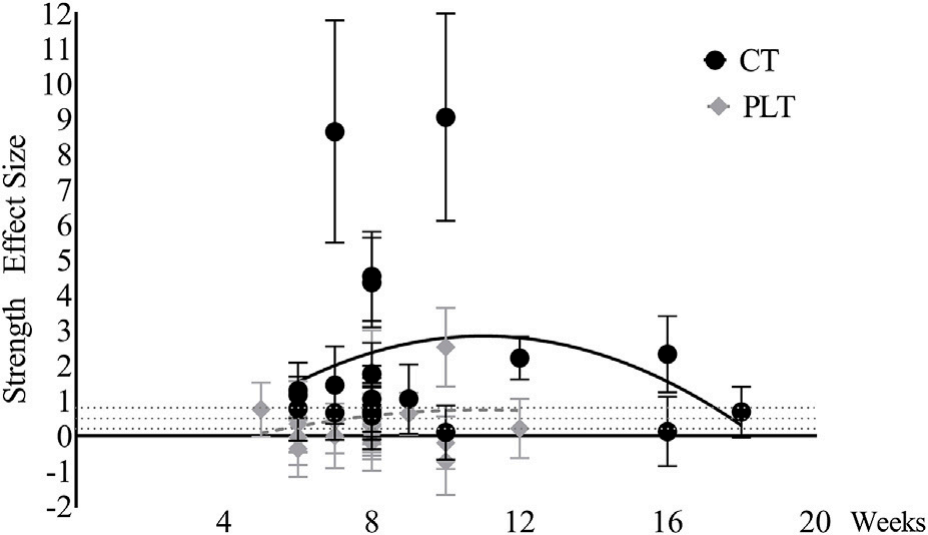
Maximum strength changes over time (CT = complex training, PLT = plyometric training).

Time effects on maximum strength. The effects of PLT on maximum strength only reached a large effect point at week 10, and the rest were below the medium effect (Figure 10). The effects of CT on maximum strength reached a large effect at week 6 and peaked at week 8, and then there was a downward trend. It should be noted that data after eight weeks were limited.

## Discussion

This is the first meta-analysis review to compare the effects of PLT vs CT on the explosive power of the lower limbs. According to research findings, during the 10-week training period, the improvement in explosive power induced by unloaded PLT was similar to that caused by CT, but that induced by loaded PLT was better. In terms of maximum strength, the effect of CT was better than that of PLT. In addition, the time effect curves of PLT and CT on explosive power were basically consistent, with an ascending trend in 10 weeks.

It is interesting to note that unloaded PLT and CT demonstrated similar effects on the explosive power of the lower limbs in 10 weeks, which is different from some previous studies (Hammami et al., 2019; Zghal et al., 2019). In theory, CT should be better than PLT in improving explosive power because CT combines the postactivation potentiation (PAP) induced by high-intensity RT and the stretch-shortening cycle in PLT. However, that is not the case. There are several explanations that could underpin these findings. One reason is that PAP did not play its due effect or PAP is just a warm-up effect. High-intensity muscle contractions stimulate the central nervous system—leading to greater motor unit recruitment of the muscle during subsequent exercise—to increase neuromuscular force, a phenomenon referred to as PAP (Hilfiker et al., 2007). PAP is an acute response and therefore time-sensitive. The optimal PAP interval time between RT and PLT is 4–10 min (Jensen and Ebben, 2003; Jo et al., 2010). Most of interval time of the included studies in this review were shorter than this standard, and inadequate interval rest may result in PAP not working. In addition, Docherty and Hodgson (2007) believed that PAP was only a warm-up effect rather than a true chronic enhancement effect. Studies by Deutsch and Lloyd (2008) and Alemdaroglu et al. (2013) confirmed Dochert’s hypothesis. Their studies found no difference in the effect of different sequences of CT (RT followed by PLT or PLT followed by RT). This indirectly indicates that PAP does not play a significant role in CT.

The review also showed that the effects of CT on maximum strength significantly higher that of PLT. This is mainly because resistance training in CT increases the maximum muscle strength. The development of explosive power is based on maximum strength (Taber et al., 2016), so CT is the better strategy for developing explosive performance more than PLT in the long term. As for why there was no difference between the two training methods in the short term, another reason may be the combined effect of maximum strength and muscle fiber type. RT can reduce the proportion of IIx muscle fibers (fast glycolysis type) (Lamas et al., 2010), which is not conducive to the improvement of RFD, namely, the improvement of explosive power. While PLT can maintain the ratio of three muscle fibers (IIx, IIa, I), which can greatly improve RFD (Zaras et al., 2013). The ratio of muscle fibers induced by CT should be between that induced by PLT and RT. Therefore, it can be concluded that the performance of fast muscles induced by CT is weaker than that induced by PLT. However, the explosive power also depends on the level of maximum strength. The improvement of maximum strength makes up for the weakness of fast muscle performance in CT. Therefore, CT and PLT have similar effects on improving explosive power in the short term. However, for long-term explosive training, the improvement of explosive power by PLT will be limited due to the limitation of maximum strength. Therefore, CT is a more appropriate choice for developing explosive power in the long term.

In the subgroup analysis of this review, the effects of loaded PLT and CT on explosive power and maximum strength were compared. Although the data of loaded PLT are limited, it could still show that the improvement of explosive power caused by loaded PLT is significantly better than that caused by CT (jump ability: 1.35 vs .85; sprint ability: −2.11 vs. −.83). The extra weight during PLT will increase the inertia and ground reaction force during the SSC eccentric stage and the resistance at the SSC concentric stage, and this high-intensity stimulation can lead to better muscular adaptation for explosive power (Negra et al., 2020). In contrast, CT only provides high concentric resistance stimulation in the RT phase, while the SSC effect in the later PLT phase was in the non-weight-bearing state. As a result, the overall muscle adaptation of CT is slightly lower than that of loaded PLT, which well explains the superiority of loaded PLT in the improvement of explosive power. However, loaded PLT also has drawbacks. The premise of the optimal effect of SSC is the rapid connection between eccentric and concentric stages. If the connection time is too long or the motion range of the joint is too large, the more elastic potential energy will be lost in the form of heat energy, thus weakening the SSC effect (Komi, 2003). At present, there is no consensus on the optimal extra load for PLT. However, according to the existing research data, no sports injury was found in loaded PLT with 0%–25% extra body weight (Rosas et al., 2016; Coratella et al., 2018; Negra et al., 2020). It is suggested that coaches should choose the PLT load carefully according to the specific situation of the athlete.

In terms of time effects, the effect curves of unloaded PLT and CT on explosive power (jump ability and sprint ability) are basically consistent and with an ascending trend in 10 weeks. This proved again unloaded PLT and CT have similar effect on explosive power and showed that more than 10 weeks of training may be beneficial for the development of explosive power. In general, Low load training while maintaining intensity is recommended during a short season because it does not create much training fatigue and affect competition performance (Zhou and Zhang, 2017). Therefore, unloaded PLT is suitable for explosive training in a short season. While long-term explosive training is based on maximum strength, CT is more suitable for annual training or long training cycles.

## Conclusion

The findings of this review suggests that unloaded PLT and CT have a similar significant effect on explosive performance (jump ability, sprint ability) in short term (within 10 weeks), but loaded PLT has a better effect. Furthermore, CT has significant beneficial effect on maximum strength compared to PLT. Therefore, we suggest applying unloaded or light-loaded PLT for explosive training in short season and applying CT in an annual or long training cycle. In addition, more than 10 weeks of training may be more beneficial for the improvement of power.

## Data Availability

The original contributions presented in the study are included in the article/Supplementary Material, further inquiries can be directed to the corresponding author.
